# Direct numerical simulation of the turbulent flow generated during a violent expiratory event

**DOI:** 10.1063/5.0042086

**Published:** 2021-03-08

**Authors:** Alexandre Fabregat, Ferran Gisbert, Anton Vernet, Som Dutta, Ketan Mittal, Jordi Pallarès

**Affiliations:** 1 Department d'Enginyeria Mecànica, Universitat Rovira i Virgili, Av. Països Catalans 26, Tarragona 43007, Spain; 2 Mechanical and Aerospace Engineering, Utah State University, 4130 Old Main Hill, Logan, Utah 84322-4130, USA; 3 Department of Mechanical Science and Engineering, University of Illinois at Urbana-Champaign, 1206 W. Green St. MC 244, Urbana, Illinois 61801, USA

## Abstract

A main route for SARS-CoV-2 (severe acute respiratory syndrome coronavirus) transmission involves airborne droplets and aerosols generated when a person talks, coughs, or sneezes. The residence time and spatial extent of these virus-laden aerosols are mainly controlled by their size and the ability of the background flow to disperse them. Therefore, a better understanding of the role played by the flow driven by respiratory events is key in estimating the ability of pathogen-laden particles to spread the infection. Here, we numerically investigate the hydrodynamics produced by a violent expiratory event resembling a mild cough. Coughs can be split into an initial jet stage during which air is expelled through mouth and a dissipative phase over which turbulence intensity decays as the puff penetrates the environment. Time-varying exhaled velocity and buoyancy due to temperature differences between the cough and the ambient air affect the overall flow dynamics. The direct numerical simulation (DNS) of an idealized isolated cough is used to characterize the jet/puff dynamics using the trajectory of the leading turbulent vortex ring and extract its topology by fitting an ellipsoid to the exhaled fluid contour. The three-dimensional structure of the simulated cough shows that the assumption of a spheroidal puff front fails to capture the observed ellipsoidal shape. Numerical results suggest that, although analytical models provide reasonable estimates of the distance traveled by the puff, trajectory predictions exhibit larger deviations from the DNS. The fully resolved hydrodynamics presented here can be used to inform new analytical models, leading to improved prediction of cough-induced pathogen-laden aerosol dispersion.

## I. INTRODUCTION

Outbreaks of infectious diseases have profoundly affected human societies on several occasions through history. The ongoing COVID-19 (coronavirus disease) pandemic is the most recent example of the extraordinary impact on health, economics, and sociopolitics of highly contagious pathogens in an increasingly globalized world. By mid-December 2020, SARS-CoV-2 has infected more than 75 × 10^6^ people worldwide killing more than 1.7 × 10^6^ of them.^1,2^

Airborne droplets and aerosols are released into the air when infected people cough, sneeze, or talk.^3,4^ These particles constitute the primary transmission route for SARS-CoV-2 (severe acute respiratory syndrome coronavirus), Measles morbillivirus, Mycobacterium tuberculosis, chickenpox, and influenza virus, among others. Understanding the airflow generated by respiratory events is key in predicting how pathogen-laden aerosols are dispersed in the environment and providing valuable information to design measures and interventions aimed at hindering airborne transmission. In the classical approach, dispersion of large aerosols or droplets (>50 *μ*m in diameter), mostly controlled by gravity, is characterized by ballistic trajectories, short residence times, and limited horizontal range. On the other end of the diameter range, small droplets or aerosols (<50 *μ*m) are mostly dispersed by the action of hydrodynamic drag, leading to the formation of aerosol clouds capable of remaining afloat over long periods of time and reaching relatively larger distances. Crucially, enhanced transport of these tiny aerosols by relatively weak background currents as those generated by heating, ventilation, and air conditioning (HVAC) has been linked to transmission between individuals separated over distances much larger than the 2 m-rule suggested by social distancing measures.^5^ While the effect of HVAC systems on far-field transmission of respiratory viruses is currently being debated and studied,^6,7^ the general agreement is that during close contacts the short-range airborne route is the dominant mode of respiratory virus transmission.^8,9^

Recent studies have used experiments^10,11^ and numerical simulations^12,13^ to analyze various aspects of the jet flow and the accompanying aerosol transport for different expiratory events including sneezing,^10^ coughing,^12^ talking, and breathing.^11^ Numerical simulations with different turbulence modeling techniques have been used to study aerosol and droplet dispersion during violent expiration events. Two of the first studies that explored the effect of factors like ambient wind^12^ and masks^14^ were conducted by Dabouk and Drikakis using the Reynolds averaged Navier–Stokes (RANS) modeling approach. Dabouk and Drikakis used the compressible multiphase mixture RANS equations and the κ−ω turbulence model to simulate the carrier expiratory jet of the cough^12^ and modeled the saliva droplets as Lagrangian particles. One of the main findings of Dabouk and Drikakis was the determination of the wind-speed influence on the airborne droplet traveled distance with droplets traveling up to 6 m for background wind speeds of 15 km h^−1^. Busco *et al.* also simulated the jet produced during a sneeze using the compressible RANS equations with the continuous phase being modeled as a mixture of air and water-vapor.^10^ The turbulence closure used in their model was κ−ϵ, and the saliva droplets were modeled as Lagrangian particles. Even though sneezing is not among the primary symptoms of Covid-19, Busco *et al.* elucidated the importance of capturing the angle of release of the expiration jet in order to accurately model the dispersion of airborne droplets.

Fontes *et al.* also used numerical simulations to investigate the fluid dynamics of a sneeze.^13^ In particular, they focused on the impact of human physiological factors (e.g., illness, stress condition, anatomy, etc.) on droplet dispersion. Fontes *et al.* modeled the expiratory jet as a gas flow under the Eulerian framework and the dispersed droplets as Lagrangian particles. Turbulence in the flow was modeled using detached eddy simulations (DES), which is based on the combination of unsteady Reynolds averaged Navier–Stokes (URANS) for the flow within the boundary layer and large eddy simulations (LESs) in the outer region. Fontes *et al.* found that the change in the nasal and buccal passages have a significant impact on the distance traveled by the droplets with nasal-obstruction resulting in an increase of up to 60%. Additionally, physical characteristics of saliva were found to change different aspects of the spray generated after the sneeze. Pendar and Pascoa conducted LES to model the dispersion of saliva droplets during violent expiratory events^15^ like coughing and sneezing. They also used an Eulerian approach for the carrier air jet and the Lagrangian approach for the droplets. The jet was modeled using the compressible Navier–Stokes equations and an LES turbulence closure where the subgrid-scale stresses were calculated using the one equation eddy-viscosity model. Pendar and Pascoa found that for a strong sneeze, large droplets (540 *μ*m in diameter) could travel up to 4 m and particles could take up to 3 s to settle down in the absence of a background flow. They also observed that bending of the head during sneezing could reduce the droplet horizontal range by 22% and face masks could further reduce the aerosol traveled distance to 0.6 m. Additionally, they concluded that droplet transport was enhanced by turbulence. This particular finding shows the importance of accurately modeling and understanding the turbulent characteristics of expiratory jets.

Wang *et al.* used alternative turbulent models based on random walk to approximate the effect of turbulence fluctuations on aerosol dispersion and study the effect of environmental factors on droplet transport and dispersion.^16^ Renzi and Clarke showed that the dynamics of the expiratory jet could be modeled by extending the theory of buoyant vortex rings.^17^ They coupled their integral model of the continuous phase with a Lagrangian particle-tracking model for the droplets and used it to explore the effect of the initial condition on the distance traveled by the droplet cloud. They also observed that the vortex in their model plays a key role in keeping droplets afloat, providing additional motivation to study in detail the turbulent structure of the expiratory jet.

A survey of the literature shows that most of the existing numerical studies focus primarily on the dispersal of the droplets due to expiration events and do not provide enough insight into the turbulent structure of the puff. Thus, we conduct a direct numerical simulation (DNS) of the flow produced by an idealized, relatively violent respiratory event resembling a mild cough, with the motivation of understanding its turbulent dynamics. The finite injection of exhaled air generates a jet that penetrates into the initially isothermal and stagnant environment. Due to the temperature difference between the injected and the ambient air, once the cough ceases, the flow transitions into a thermal puff that bends in the vertical as turbulence decays due to dissipation.

Scorer^18^ experimentally investigated the evolution of isolated masses of buoyant fluid or thermals by releasing finite volumes of heavy solutions into a water tank. The results were used to validate an analytical model in which the isolated mass of buoyant fluid kept its linear momentum by decelerating as its volume grew through entrainment of lighter ambient fluid. The mean value of the entrainment coefficient, relating the thermal radius *r* and the vertically traveled distance form the source *y*, i.e., r=αy, was found to be α=0.25 (or n=4=1/α in the original paper). The mean prefactor *η* in the relation between radius and volume of the thermal, $V=\eta \;r^{3}$, was estimated^18^ to be *η* = 3.

Puffs, occurring when the isolated mass of fluid has some initial momentum, were investigated by Richards^19^ who experimentally produced both axial (point source) and cylindrical (line source) thermals reporting estimations for *α* between 0.13 and 0.53. Experiments on axial thermal puffs by Richards^20^ were used to derive a relation between the traveled distance *z* and time *t* of the form $z^{4}=Cn^{3}Mgt^{2}/\rho_{\infty }$, where *C* is a constant, *M* is the mass excess, *g* is the gravity acceleration, and $\rho_{\infty }$ is the ambient density.

By directing the flow with some angle *β*_0_ with respect to gravity aligned with the *y* direction, Richards^21^ derived a model for inclined puffs of constant total buoyancy by solving the momentum equations $P_{z}={\left|P\right|}_{0}\;\text{cos}\;(\beta_{0})$ and $P_{y}={\left|P\right|}_{0}\;\text{sin}\;(\beta_{0})+\left|Mg\right|t$, where $\vec{P}$ is the puff impulse. Aimed to better understand puffs and thermals in the atmosphere, Scorer^22^ reported a parameter-free model for predicting the front location, velocity, and radius of a turbulent puff.

Using the analytical solution for inclined thermal puffs derived by Richards,^21^ Bourouiba, Dehandschoewercker, and Bush^23^ investigated the flow produced by violent expiratory events by comparing the model predictions with experimental results obtained by horizontally injecting particle laden fresh water payloads into a tank filled with salty water. The puff trajectory and shape were obtained by tracking the frontal region and fitting an spheroid (two identical semi-axes) for successive snapshots. In contrast to ellipsoidal shapes characterized by three independent semi-axis lengths, the semi-axis most aligned with the vertical, used to estimate the puff radius *r*, was assumed to be equal in length to the one in the spanwise direction.

In this work, a similar approach is used to elucidate the three-dimensional trajectory and shape of a puff produced by a numerical cough. Fully resolved results on the flow hydrodynamics suggest that, along with the self-similar hypothesis in the Scorer^22^ and Bourouiba, Dehandschoewercker, and Bush^23^ models and the transient behavior of a cough air injection, the spheroidal assumption may contribute to explain the observed differences between the experimental and analytical attempts to characterize the flow produced by a violent expiratory event and the DNS predictions. Given the major role played by the background fluid hydrodynamics in the aerosol dispersion, the new insight presented here can be used to improve our current predictive capabilities of infection risk by airborne-transmitted pathogens.

Although Direct Numerical Simulations have been carried out in the past,^24^ to our knowledge, this is the first time that details on the temporal evolution of puff centroid, entrainment coefficient, and front topology have been reported.

## II. PHYSICAL AND MATHEMATICAL MODEL

A mild cough is modeled as a finite injection of air into an initially isothermal and quiescent environment. Exhaled and ambient air temperatures are $T_{0}=34$°C and $T_{\infty }=15$°C, respectively. The transient nature of the cough flow is captured^25^ by imposing an inlet transient velocity $w_{0}(t)$ that ramps up linearly from zero to the peak velocity $w_{m}=4.8$ m s^−1^ at $t_{m}=0.15$ s and then ramps down linearly to zero at $t_{c}=0.4$ s, i.e.,

$$w_{0}(t)=\{\begin{array}{cc} \frac{w_{m}}{t_{m}}t, & 0\leq t<t_{m}\\ w_{m}-\frac{w_{m}}{t_{c}-t_{m}}\left(t-t_{m}\right), & t_{m}\leq t\leq t_{c}\\ 0, & t>t_{c}. \end{array}$$ (1)

Thus, air flow generates an accelerating ($0\leq t<t_{m}$) and a decelerating ($t_{m}\leq t\leq t_{c}$) jet that eventually evolves into a thermal puff when injection ceases for $t>t_{c}$.

Air thermal conductivity, kinematic viscosity, thermal diffusion coefficient, and thermal expansion coefficient evaluated at $T_{a}=(T_{0}+T_{\infty })/2$ are assumed to remain constant at $k_{f}=0.026\;m^{-1}K^{-1}$, $\nu =1.6\times 10^{-5}m^{2}s^{-1}$, $\alpha =2.24\times 10^{-5}m^{2}s^{-1}$, and $\beta =0.003\;47\;K^{-1}$, respectively. Density variations with temperature are considered only in the buoyancy term of the vertical momentum equation according to the Boussinesq approximation [with $\rho_{a}(T_{a})=1.22\;\text{kg}\;m^{-3}$]. The air exit is modelled^25^ as a cylindrical pipe of diameter *d*  =  0.02 m and length $H_{p}=0.04$ m. The subject is assumed to remain steady with a pitch angle perpendicular to gravity at all times. Under the above stated conditions, the airflow during the cough can be modeled as an incompressible flow.^26^

The conservation equations for mass, momentum, and energy for incompressible flows can be written as

$$\frac{\partial u_{i}}{\partial x_{i}}=0,$$ (2)

$$\frac{\partial u_{i}}{\partial t}+u_{j}\frac{\partial u_{i}}{\partial x_{j}}=-\frac{1}{\rho_{a}}\frac{\partial p}{\partial x_{i}}+\nu \frac{\partial^{2}u_{i}}{\partial x_{j}\partial x_{j}}+g\beta (T-T_{\infty })\;\delta_{i2},$$ (3)

$$\frac{\partial T}{\partial t}+u_{j}\frac{\partial T}{\partial x_{j}}=\alpha \frac{\partial^{2}T}{\partial x_{j}\partial x_{j}},$$ (4)

where *t* is the time, *p* is the pressure, *T* is the temperature, *δ_ij_* is the Kronecker delta, and $u_{i}=(u,v,w)$ is the velocity field with coordinates $x_{i}=(x,y,z)$ in the spanwise, gravity-aligned, and streamwise directions. Using the exit diameter *d*, the inlet peak velocity $w_{m}$, and the maximum temperature difference $\Delta T=T_{0}-T_{\infty }=19{}^{\circ}C$ as space, velocity, and temperature scales, the Reynolds, Richardson, and Péclet numbers are $Re=w_{m}d/\nu =6000,\;Ri=g\beta \Delta Td/{w_{m}}^{2}=5.61\times 10^{-4}$, and $Pe=w_{m}d/\alpha =4200$, respectively. The gravity acceleration is $g\;\delta_{i2}=-9.8\;m\;s^{-2}$, and the temperature perturbation is defined as $\tilde{\theta }=(T-T_{\infty })/\Delta T$. Note that the tilde symbol is reserved for non-dimensional variables. The variation of the physical properties with the water vapor concentration is neglected. This approximation is reasonable under the current conditions considered. For example, for exhaled air at *T* = 34 °C and relative humidity RH=85%,^27^ $\rho =1.130\;\text{kg}\;m^{-3}$, and for ambient conditions at *T* = 15 °C and $RH=65\%,\;\rho =1.220\;\text{kg}\;m^{-3}$. However, for air at *T* = 34 °C and $RH=65\%,\;\rho =1.134\;\text{kg}\;m^{-3}$.

**FIG. 1.**
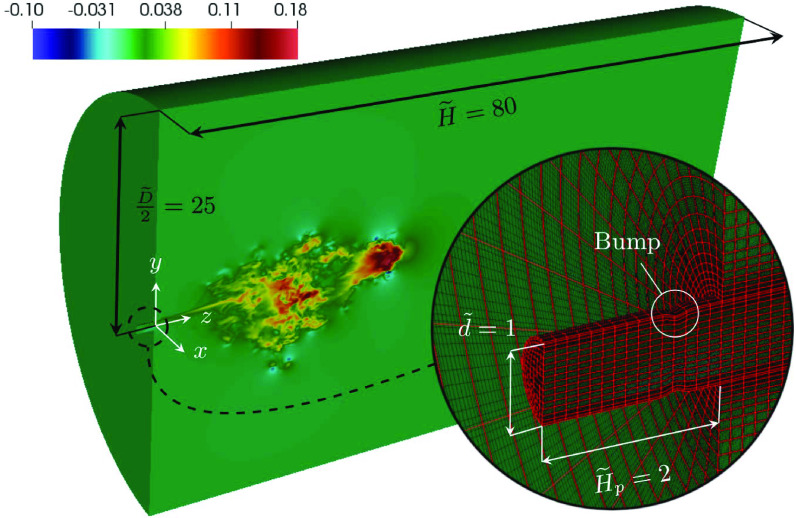
Section of the computational domain showing an instantaneous *w* velocity component field at *t* = 0.75 s. The inset shows the spectral element mesh (in red) and the LGL nodes (in black).

The computational domain dimensions, the coordinate system, and the mesh details are illustrated in Fig. 1 using non-dimensional variables. The inlet boundary conditions in the cylindrical injection section of diameter ($\tilde{d}=1$) and length (${\tilde{H}}_{p}=2$) are ${\left(\tilde{u},\tilde{v},\tilde{w}\right)|}_{\tilde{x},\tilde{y},\tilde{z}=-2,\tilde{t}}=(0,0,{\tilde{w}}_{0}(\tilde{t}))$ and ${\tilde{\theta }|}_{\tilde{x},\tilde{y},\tilde{z}=-2,\tilde{t}}=1$, with boundary conditions for the outer walls set to no-slip and adiabatic. An annular indent of the Gaussian profile with the center at ${\tilde{z}}_{d}=-1/2$, a depth of ${\tilde{h}}_{d}=0.05$, and a width of ${\tilde{\sigma }}_{d}^{2}=0.01$ has been used to mimic the complicated passage of exhaled air across the human mouth, cause boundary layer separation, and facilitate the transition to turbulence. The main domain consists of a cylinder of diameter $\tilde{D}=D/d=50$ and length $\tilde{H}=H/d=80$. Outflow boundary conditions have been imposed at *z* =* H* and at the outer cylindrical wall of radius R=D/2, which have also been considered adiabatic. These dimensions ensure that the cough flow does not interact with the boundaries over the temporal extent of the simulation. Initial conditions over the entire computational domain are $\tilde{u}=\tilde{v}=\tilde{w}=\tilde{\theta }=0$.

The results presented here have been obtained using Nek5000,^28^ an open-source high-order spectral element method (HO-SEM)-based solver for the incompressible Navier–Stokes equations (INSEs). The SEM is a high-order weighted residual method that combines the geometric flexibility of finite elements (Ω is decomposed into *K* smaller elements) with the rapid convergence of spectral methods. The basis functions in SEM are *N*th-order tensor-product Lagrange polynomials on the Gauss–Lobatto–Legendre (GLL) quadrature points inside each element, which lead to fast operator evaluation and low operator storage cost.^29^ In the SEM-based solver Nek5000, the unsteady INSEs are solved in velocity-pressure form using semi-implicit BDF*k*/EXT*k* timestepping in which the time derivative is approximated by a *k*th-order backward difference formula (BDF*k*), the nonlinear terms (and other forcing) are treated with a *k*th-order extrapolation (EXT*k*), and the viscous and pressure terms are treated implicitly. This approach leads to a linear unsteady Stokes problem to be solved at each time step, which comprises a Helmholtz equation for each component of velocity (and temperature/scalar) and a Poisson equation for pressure (see, e.g., Sec. 2.2 in the study by Mittal^30^) Based on this approach, SEM has proven to be well suited for turbulent flows.^31–33^

For the current DNS, we model the domain using *K* = 215 820 elements with *N* = 11th order polynomials for the solution. The total number of mesh nodes is $K\times {\left(N+1\right)}^{3}\approx 370$ × 10^6^. The simulation has been run on 20 nodes of a Central Processing Unit (CPU) cluster, interconnected with a 100 Gb/s Infiniband network. Each node contains two Intel Platinum 8168 CPUs with 24 cores each. The average CPU time per time step is around 5 s. The simulation took $5.19\times 10^{5}$ CPU h to reach *t* = 1.68 s.

Glezer and Coles^34^ estimated the production of turbulent kinetic energy of a self-similar momentum puff using laboratory measurements. They reported typical values of production of turbulent kinetic energy around $\Pi =100{\left(I/\rho_{f}\;t^{5}\right)}^{1/2}$ within the vortex ring, where *I* is the puff impulse ($I=6\times 10^{-4}$ N s in the present DNS study). Assuming that production equals dissipation, the ratio between the Kolmogorov length scale *η_K_* and the exit diameter is $\eta_{K}/d\approx 0.01\;t^{5/8}$. This result is compatible with the estimation usually reported for DNSs of jets based on the measurements of Panchapakesan and Lumley^35^ (see, for example, the study by Boersma, Brethouwer, and Nieuwstadt^36^) According to these authors, for the present Reynolds number based on the maximum velocity during the cough, $\eta_{K}/d\approx 6\times 10^{-4}x/d$. The simulation shows that the initially laminar jet becomes completely turbulent, with fine scale activity, at *t* = 0.3 s ($\tilde{t}=72$). At this time, the puff is approximately at *x* = 15 ($\tilde{x}=15d$) and the estimations of the non-dimensional Kolmogorov length scale are $5\times 10^{-3}$ and $10^{-2}$ according to the criteria based on the measurements of Glezer and Coles^34^ and Panchapakesan and Lumley,^35^ respectively. The grid sizes at the jet axis at this position are $\Delta \tilde{x}=\Delta \tilde{y}\approx 0.009$ and $\Delta \tilde{z}\approx 0.04$, which are of the same order of magnitude as the estimations.

**FIG. 2.**
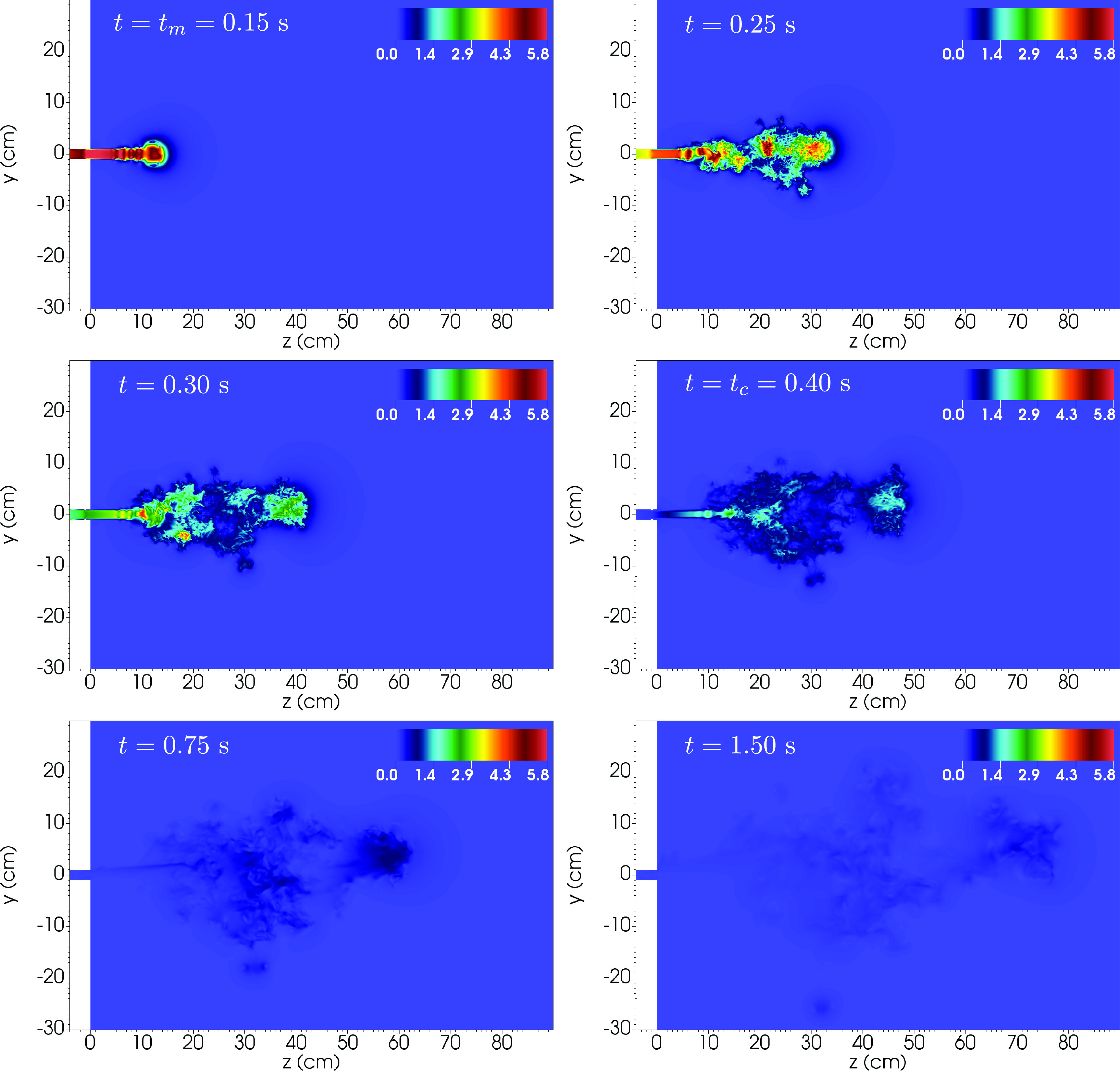
Detail of the slice at *x* = 0 of the velocity magnitude field (in m s^−1^) at t=0.15,0.25,0.30,0.40,0.75,1.5 s. Note that $t=t_{m}=0.15$ s and $t=t_{c}=0.40$ s correspond to the peak and cough ending times, respectively.

**FIG. 3.**
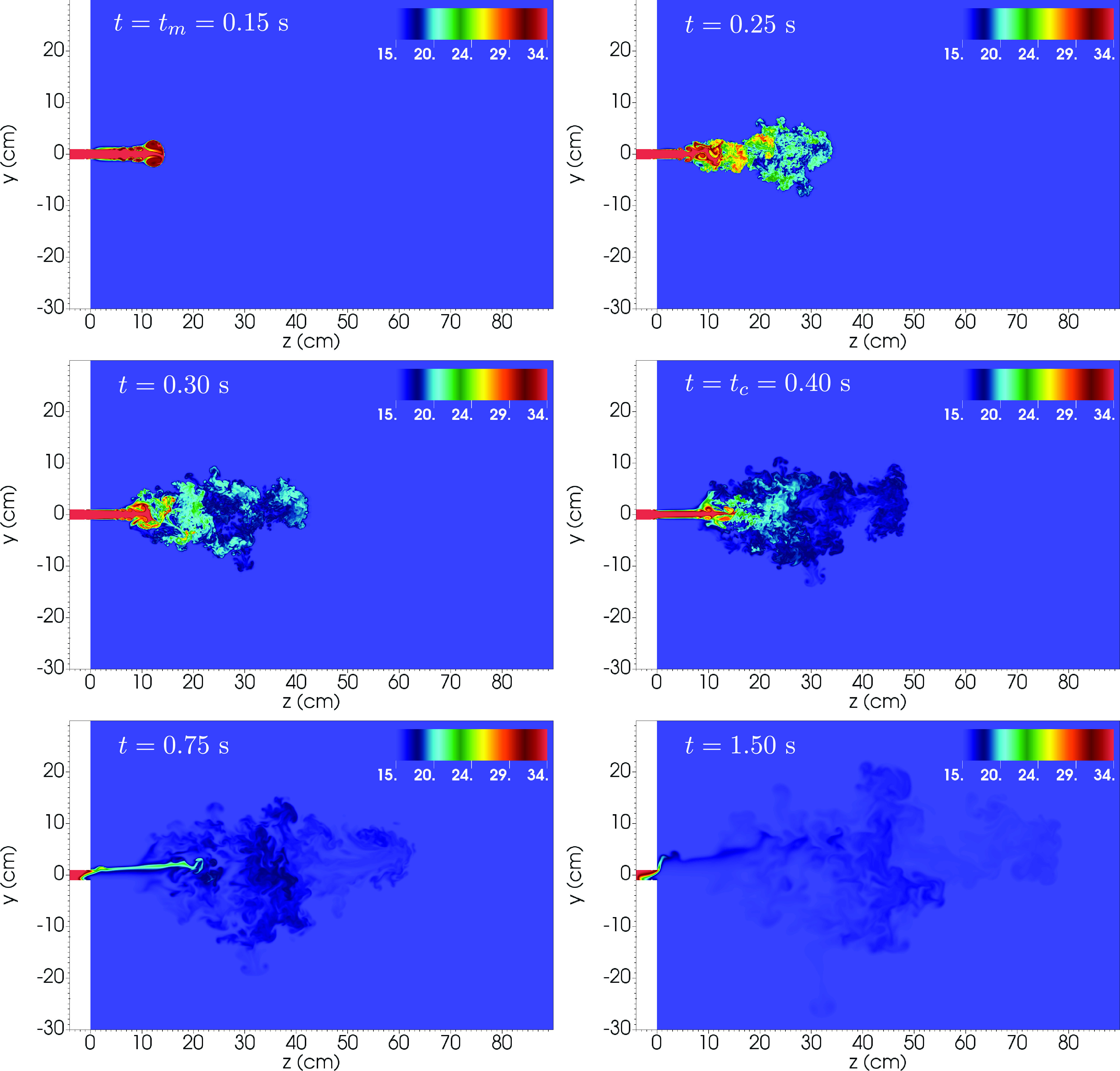
Detail of the slice at *x* = 0 of the temperature field (in °C) at t=0.15,0.25,0.30,0.40,0.75,1.5 s. Note that $t=t_{m}=0.15$ s and $t=t_{c}=0.40$ s correspond to the peak and cough ending times, respectively.

The flow hydrodynamics is illustrated in Figs. 2 and 3 that show a detail of the *x* = 0 plane for the instantaneous velocity magnitude (in m s^−1^) and temperature (in °C) at six different times, including the instants of peak velocity ($t=t_{m}=0.15$ s) and the end of the cough ($t=t_{c}=0.40$ s), respectively.

## III. THEORETICAL BACKGROUND

The distance traveled by a horizontal buoyant puff released in quiescent, neutral ambience can be written as^21^

$$\begin{array}{c} s(t)=\{\frac{4}{\eta \;\alpha^{3}\rho_{f}}\frac{P_{0}^{2}}{2B_{0}}[\frac{B_{0}\;t}{P_{0}}\sqrt{1+{\left(\frac{B_{0}\;t}{P_{0}}\right)}^{2}}\\ +{\;\text{ln}\;\left(\frac{B_{0}\;t}{P_{0}}+\sqrt{1+{\left(\frac{B_{0}\;t}{P_{0}}\right)}^{2}}\right)]\}}^{1/4}, \end{array}$$ (5)

where $P_{0}=\rho_{f}\;V_{0}\;\bar{w}$ and $B_{0}=\Delta \rho \;V_{0}\;g$ are the initial linear momentum and the initial buoyancy of the puff with initial ejected volume *V*_0_ and average velocity $\bar{w}$, respectively, *α* is the entrainment coefficient, and *η* is a constant that depends on the shape of the puff. Equation (5) assumes that the velocity distributions inside the puff are similar and that the momentum of the puff remains constant during its dispersion. The linear momentum is conserved by the entrainment of the quiescent ambient fluid, resulting in a puff that increases in mass and decelerates as it penetrates into the ambient. In inclined puffs and thermals, where the puff may bend due to buoyancy effects, the radial spread of the puff can be written^23^ as r(t)=α s(t).

Previously reported^18,19^ values of *α* for thermals are 0.25 and between 0.13 and 0.53. In their experiments, Bourouiba, Dehandschoewercker, and Bush^23^ found smaller values ranging between 0.09≤α≤0.18 for the jet stage and 0.015≤α≤0.037 for the horizontal buoyant puff phase.

The behavior of a horizontal buoyant puff released with an initial momentum during a short initial period of time ($0\leq t\leq t_{j,\mathrm{end}}$) can be understood as an initial turbulent jet that evolves to a puff.^23,37^ During the jet stage ($0\leq t\leq t_{j,\mathrm{end}}$) the axial location *z_j_*, the axial velocity *W_j_* and the radial spread *R_j_* are^38^

$$z_{j}(t)={\left(\frac{12}{K}\right)}^{1/2}{\left(W_{j0}R_{j0}\right)}^{1/2}t^{1/2},$$ (6)

$$W_{j}(t)=\frac{6W_{j0}R_{j0}}{Kz_{j}(t)},$$ (7)

$$R_{j}(t)=R_{j0}+\frac{\left(z_{j}(t)-z_{j0}\right)}{n_{j}}.$$ (8)

For the puff stage ($t\geq t_{j,\mathrm{end}}$), the axial location *z_p_* can be expressed as^22^

$$z_{p}(t)=z_{j,\mathrm{end}}+{\left(4W_{j,\mathrm{end}}R_{j,\mathrm{end}}^{3}n_{p}^{3}\right)}^{1/4}\left(t^{1/4}-t_{j,\mathrm{end}}^{1/4}\right).$$ (9)

The subscripts 0, *j*, and *p* indicate initial state, jet, and puff, respectively. In Eqs. (6) and (7), *K* = 0.457 is a constant for jets,^38^ $R_{j0}$ is the radius of the orifice of the jet, and $W_{j0}$ is the exit velocity of the jet. $z_{j0}$ in Eq. (8) is the position of the virtual origin of the jet, which can be neglected under the conditions of the simulation. The parameter *n* in the jet and puff equations defines the angle of spread, *θ*, n=1/ tan θ=x/r. For jets,^22^ $\theta_{j}\approx 11.3{}^{\circ}$ and *n_j_* = 5 and for puffs,^22^ $\theta_{p}\approx 14.0{}^{\circ}$ and *n_p_* = 4.

## IV. RESULTS

### A. Puff characterization

The trajectory followed by the thermal puff is obtained following an analogous approach to that used by Bourouiba, Dehandschoewercker, and Bush^23^ who determined the ellipse that properly enclosed the front region of the dyed puff in consecutive photographic snapshots. The longest of the semi-axes, mostly perpendicular to *z* along the entire experiment, was used as a measure of the puff radius *r*.

For the present numerical results, each numerical snapshot (k=1…224) of the instantaneous $\tilde{\theta }$ field is first interpolated into a Cartesian grid with similar average resolution to that used in the computational mesh, then integrated along *x*, and finally binarized using an indicator function *i_k_* with a prescribed tolerance of $\varepsilon =10^{-3}$, i.e.,

$$i_{k}=\{\begin{array}{cc} 1, & {\left\langle {\tilde{\theta }}_{k}\right\rangle }_{x}\geq \varepsilon \\ 0, & {\left\langle {\tilde{\theta }}_{k}\right\rangle }_{x}<\varepsilon . \end{array}$$ (10)

The instantaneous temperature integrated along *x*, ${\left\langle \tilde{\theta }\right\rangle }_{x}$, is thought to mimic the photographic images taken during the experiments by Bourouiba, Dehandschoewercker, and Bush.^23^

Using the contour of *i_k_*, the puff front is fitted by a 3D ellipsoid with the centroid $\left(c_{x},c_{y},c_{z}\right)$ and semi-axes $\left(\sigma_{1},\sigma_{2},\sigma_{3}\right)$.^39^ To mimic the analysis in Ref. 23, we define *r* as the longest projected semi-axis on the plane *y* – *z*.

**FIG. 4.**
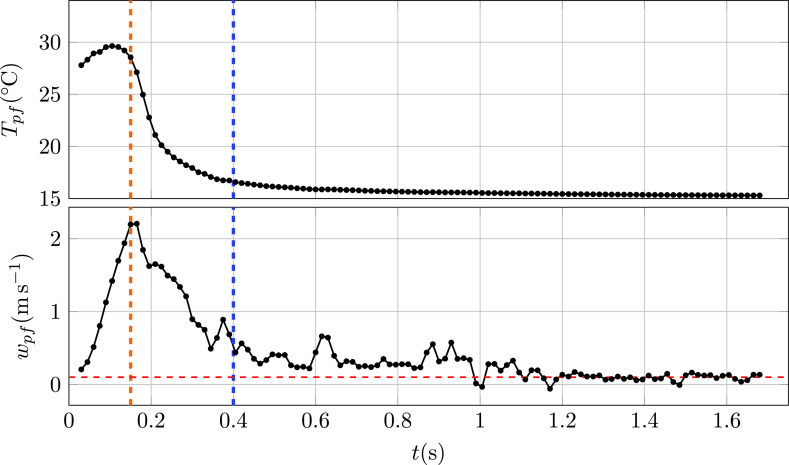
Puff front temperature and vertical velocity.

The puff front temperature and vertical velocity are shown in the top and bottom panels of Fig. 4, respectively. Results suggest that, after the end of the cough event (marked by the vertical dashed blue line), temperature within the puff front decays exponentially as turbulent mixing entrains fresh fluid. The horizontal velocity in the puff front peaks at the same time the injection does (vertical dashed orange line) to later decay to values close to that typical of indoors conditions^40^ of 0.1 m s^−1^ indicated by the horizontal red dashed line.

### B. Estimation of *η*

In the study by Bourouiba, Dehandschoewercker, and Bush,^23^ the volume of the puff front is defined as $V_{p}=\eta r^{3}$, where *r* is the longest semi-axis of the fitted ellipse projected in plane *y* – *z* and *η* is a shape factor that takes the value of $\frac{4}{3}\pi$ for spherical puffs. Equating *V_p_* to the volume of an ellipsoid, $V_{s}=\frac{4\pi }{3}\sigma_{1}\sigma_{2}\sigma_{3}$, *η* can be estimated as

$$\eta =\frac{4\pi }{3}\frac{\sigma_{1}\sigma_{2}\sigma_{3}}{r^{3}}.$$ (11)

### C. Puff trajectory

**FIG. 5.**
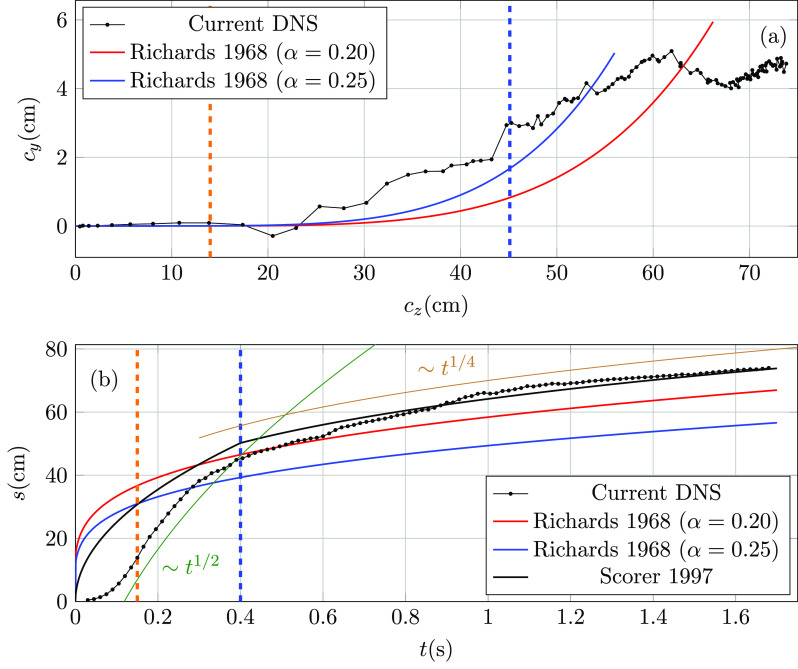
Panel (a): Puff front trajectory ellipsoid centroid from the DNS (thin dotted black) and the Richards^21^ model for α=0.20 (red) and α=0.25 (blue). Panel (b): Temporal evolution of the centroid traveled distance from the DNS (thin dotted black), the Richards^21^ model for α=0.20 (red) and α=0.25 (blue), and the Scorer^22^ model (solid black). Vertical dashed orange and blue lines indicate the peak velocity and cough end times, respectively. Curves for $t^{1/2}$ and $t^{1/4}$ added for reference.

The trajectory of the puff extracted from the temporal evolution of the ellipsoid centroid and the prediction from the model of Richards^21^ expressed in Eq. (5) are shown in Fig. 5(a) for two values of the entrainment coefficient. While the model prediction exhibits marginal puff deflection over the duration of the jet (end of cough indicated by the blue vertical dashed line), the numerical results suggest that deflection starts soon after the peak velocity has been reached (orange vertical dashed line). Note that the initial momentum (*P*_0_) in Eq. (5) has been calculated with the time averaged velocity $\bar{w}=w_{m}/2$. Contrary to the models that assume a constant velocity during the injection, the decrease in the inlet momentum, as injection velocity ramps down, allows buoyancy forces to start deflecting the jet after reaching the peak velocity. Once the cough has finished, the thermal puff continues to rise, while temperature mixes and turbulence decays due to dissipation. The deflection rate of the plume eventually decreases for $c_{z}≳$ 62 cm. This behavior cannot be reproduced by the model that predicts a continuous increase in the amount of entrained fluid into the puff.

The temporal evolution of the thermal puff traveled distance *s* is shown in Fig. 5(b) that also includes the models of Richards^21^ given in Eq. (5) and Scorer^22^ given in Eqs. (6)–(9). While models predict a monotonic growth in *s*, the numerical results exhibit an acceleration stage during the laminar jet increase over $0<t\leq t_{m}$ (orange vertical dashed line). Once the inlet velocity starts to ramp down, the *s* curve follows a $t^{1/2}$ [Eq. (6)] trend up to the end of the cough (blue vertical dashed line). The differences in the initial stages of the cough, explained by the initial laminar regime of the jet and the transient nature of the injection, decrease once the thermal puff stage is reached and *s* exhibits a $t^{1/4}$ growth rate [Eq. (9)]. While larger *α* values result in better predictions of the centroid trajectory, the faster rate of entrainment leads to underpredicted traveled distance *s*.

### D. Puff front growth and topology

**FIG. 6.**
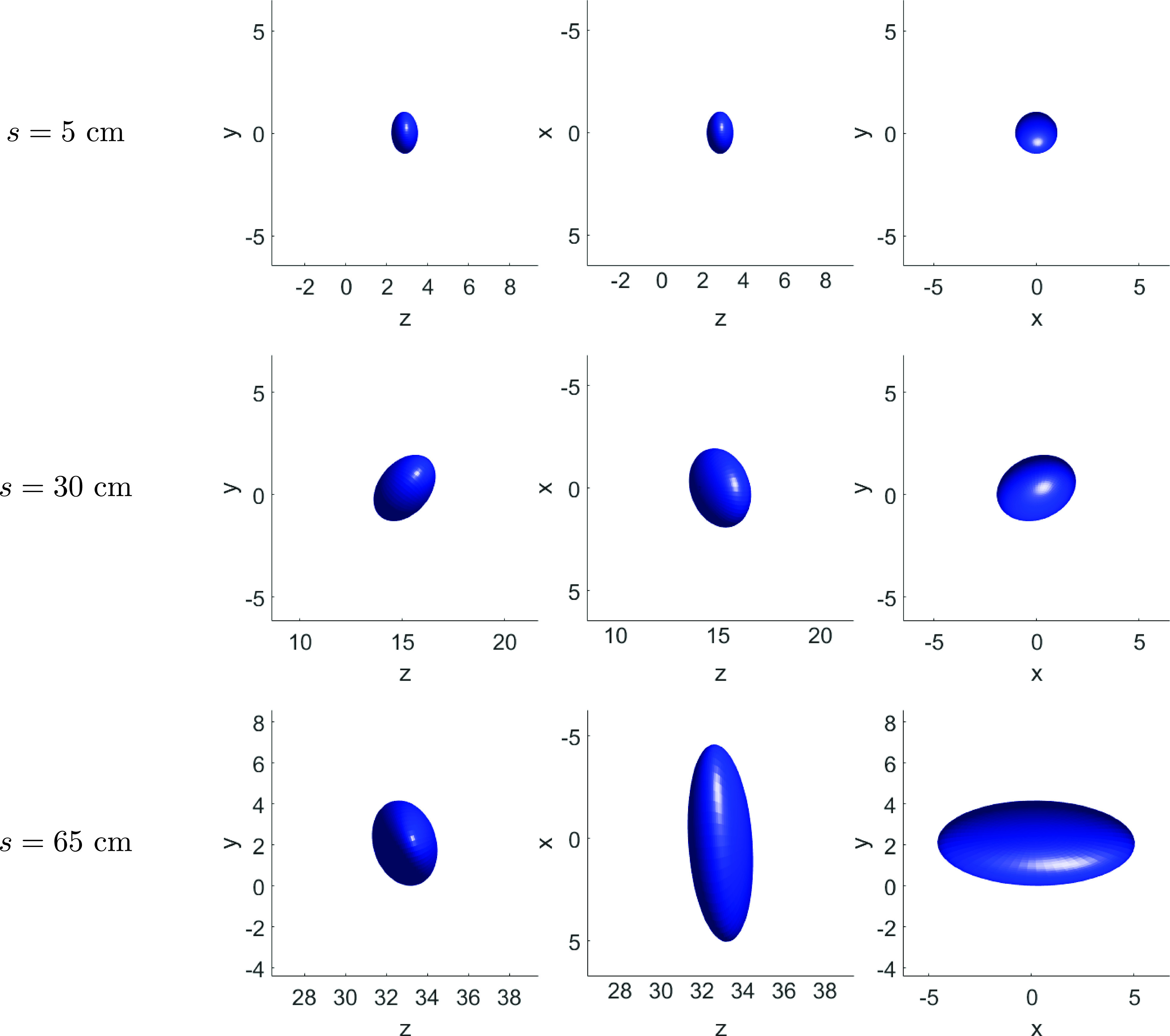
The three projections on *z* – *y*, *z* – *x*, and *x* – *y* planes of the best-fitting ellipsoid at three different values of traveled distance s= 5, 30, and 65 cm corresponding to times t≈ 0.1, 0.24, and 1.0 s. All axes in cm.

The three projection views of the best-fitting ellipsoid to the puff front at s= 5, 30, and 65 cm are shown in Fig. 6. Results suggest that, as the puff penetrates in the environment, the fastest growing semi-axis in this flow realization is the one quasi-parallel to the *x* direction.

**FIG. 7.**
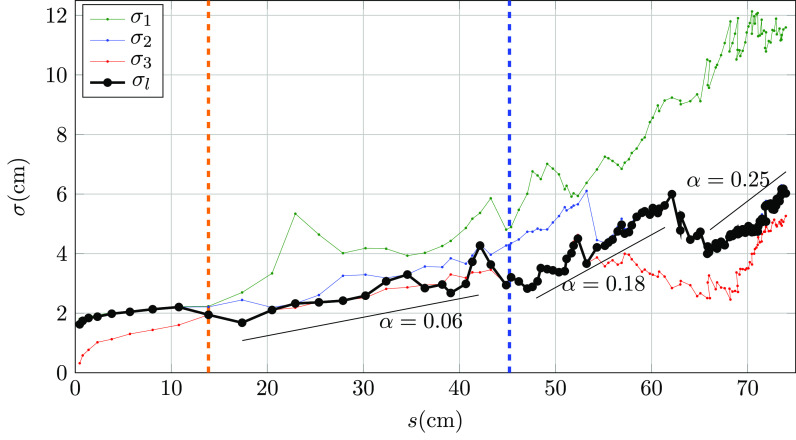
Temporal evolution of the 3D ellipsoid semi-axes *σ_i_* (red, blue, and green) and the largest projected semi-axis on the *z* – *y* plane *σ_l_* (black) with local values of the entrainment coefficient *α* for the present DNS realization. Peak and end cough times are shown as vertical orange and blue dashed lines, respectively.

**FIG. 8.**
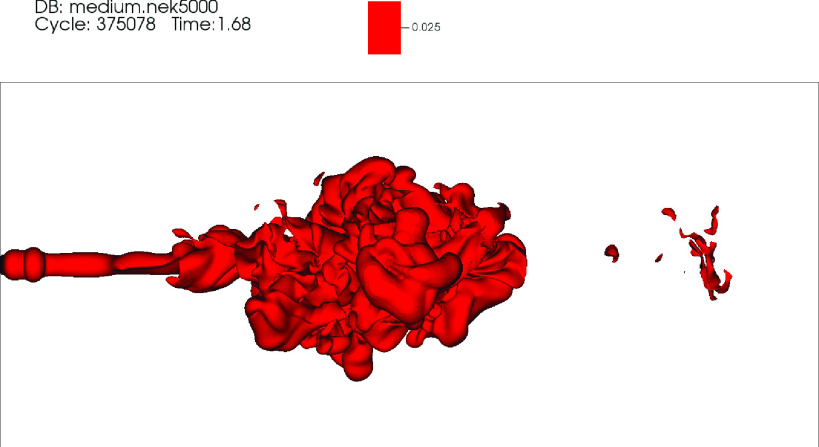
Video 1: Top *x* – *z* view showing the temporal evolution of the θ=0.025 isosurface for the DNS of a mild cough. Time is in seconds. Multimedia view: http://dx.doi.org/10.1063/5.0042086.1
10.1063/5.0042086.1

**FIG. 9.**
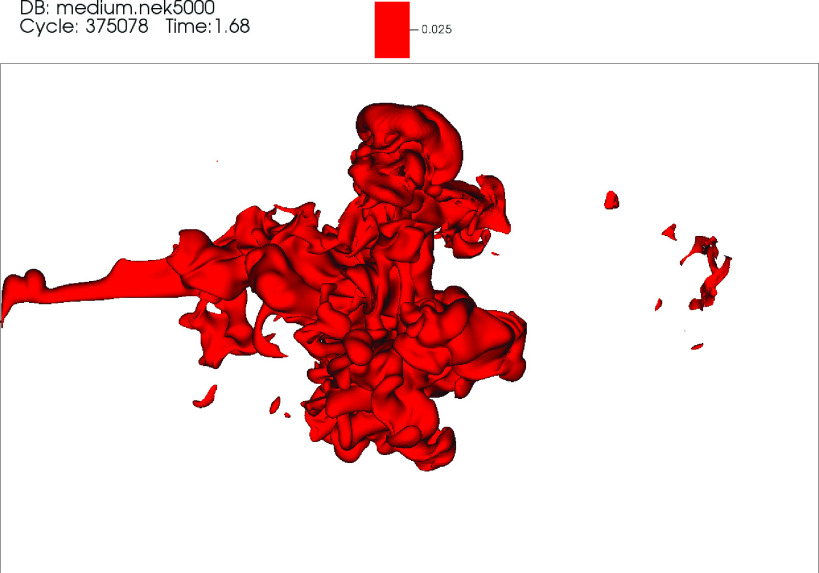
Video 2: Side *x* – *y* view showing the temporal evolution of the θ=0.025 isosurface for the DNS of a mild cough. Time is in seconds. Multimedia view: http://dx.doi.org/10.1063/5.0042086.2
10.1063/5.0042086.2

**FIG. 10.**
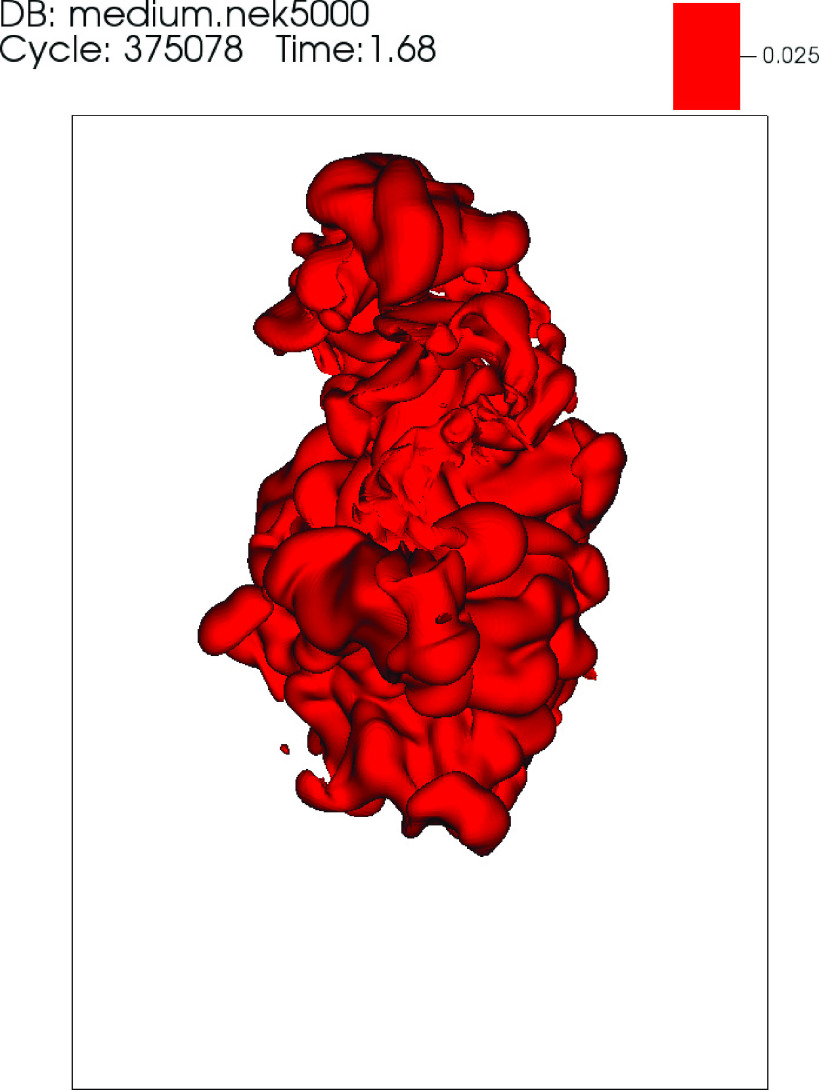
Video 3: Front *y* – *z* view showing the temporal evolution of the θ=0.025 isosurface for the DNS of a mild cough. Time is in seconds. Multimedia view: http://dx.doi.org/10.1063/5.0042086.3
10.1063/5.0042086.3

Details of the temporal evolution of the puff front topology are provided in Fig. 7. Results for the three semi-axes of the ellipsoid suggest that, after reaching the peak velocity, the puff topology starts to depart from the spheroidal shape assumed by Bourouiba, Dehandschoewercker, and Bush.^23^ The opposite thermal vertical structure across the puff leads to enhanced turbulent vertical mixing in the upper shear layer and diminished mixing in the lower one. As a result, the horizontal location of the top part of the front is delayed and the *y* – *z* projected ellipsoid appears rotated in the counterclockwise direction for $t>t_{c}$. Animations showing the temporal evolution of the $\tilde{\theta }=0.025$ isosurface in the top (*z* – *x* projection), side (*z* – *y* projection), and front (*x* – *y* projection) views are included in Figs. 8–10, respectively (Multimedia view).

The growth rate of the puff radius *r*, defined as the longest semi-axis of the projected ellipsoid on the *y* – *z* plane (black line), clearly exhibits different regimes over the duration of the cough. The results indicate that the decelerating jet exhibits a relatively small entrainment coefficient of α=0.06. Once the cough ceases, the puff seems to be characterized by intermittent episodes of rapid entrainment with coefficient values ranging over 0.18<α<0.25. These events can also be seen in the measurements of Bourouiba, Dehandschoewercker, and Bush^23^ [see, for example, their Fig. 9(b)]. A value of α=0.2 would, therefore, overpredict the cough growth during its early stage and underpredict it, at least locally, once the turbulent puff is totally developed.

**FIG. 11.**
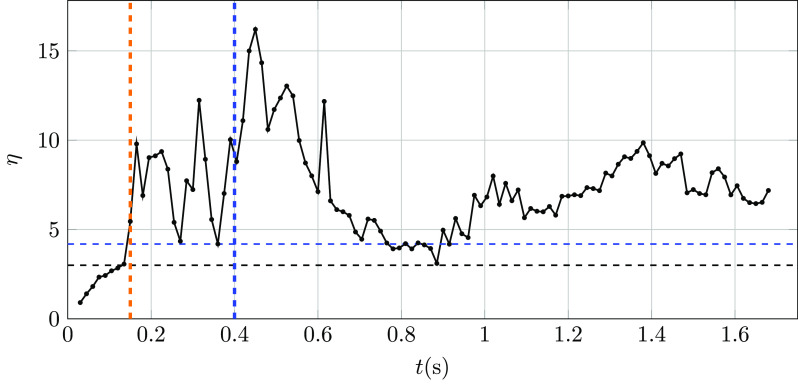
Temporal evolution of *η* as defined in Eq. (11) for the present DNS realization. Orange and blue vertical dashed lines indicate the peak and end of cough times, respectively. The value *η* = 3 suggested by Bourouiba, Dehandschoewercker, and Bush^23^ and η=4π/3 are shown as a horizontal black and blue dashed lines, respectively.

The temporal evolution of the shape factor *η* defined in Eq. (11) is shown in Fig. 11. The pronounced disparity in the length of the three ellipsoid semi-axes suggests that the puff front shape notably differs from that previously assumed by both Richards^20^ and Bourouiba,^23^ which lead to underestimation of the puff volume growth rate. Numerical results suggest that after the peak velocity time, the initially slender puff rapidly grows until reaching its largest volume soon after the end of the cough. Near the end of the simulation at t≈1.65 s, the puff volume shrinks to values of *η* approximately twice the value used in the study by Bourouiba, Dehandschoewercker, and Bush.^23^

## V. DISCUSSION

Turbulence is a chaotic process in nature, and therefore, the sole DNS realization of the flow produced by a mild cough reported here does not provide information regarding turbulence properties. In addition, the flow configuration assumed a simplified respiratory track geometry, a stagnant and homogeneous environment, and an ambient air temperature typical of outdoors conditions at mid latitudes. Although changes in the “mouth” diameter and exit velocity would affect the Reynolds number, the variability of this parameter over the typical range observed for violent expiratory events including coughs and sneezes is expected to be relatively limited and, therefore, does not significantly change the integral quantities of interest, namely, the puff horizontal penetration and lateral growth rate. Regarding the ambient air temperature, a larger value of $T_{\infty }$ closer to typical indoors conditions should result in weakened vertical puff deflection. With a vertical displacement of approximately 0.05 cm per horizontally traveled cm, an increase in the overall horizontal penetration in warmer ambient conditions is expected to be modest.

Current effort is directed to explore the effects of potentially key variables capable of significantly impacting the flow hydrodynamics of expiratory events. On the one hand, the rapid kinetic energy dissipation observed once the exhalation ceases in the present results suggests that, for times as short as *t* = 1 s after the cough onset, the puff average velocity decays to values typical of indoor conditions. This suggests that background currents produced by heating, ventilation, and air conditioning may play a significant role in the late stages of the thermal puff evolution. Similarly, ambient thermal stratification may notably change the turbulent dissipation by suppressing the vertical momentum and heat transport, which, in turn, could affect the horizontal, drag-dominated dispersion of pathogen-laden aerosols. Finally, milder exhalation events including talking or signing are suspected to exhibit significantly different dispersion patterns due to their sustained and transient nature.

## VI. CONCLUSIONS

The resolved numerical simulation of an idealized violent expiratory event provides unprecedented details on the temporal and spatial evolution of the jet and thermal puff produced by a mild cough. New insight into the flow hydrodynamics generated by a mild cough could help in developing new models for dispersion of pathogen-laden aerosols that are responsible for airborne transmission of infectious diseases. The DNS results presented here could be used to validate/improve reduced order models that can then be used in physics-based transmission-models.^41^

While the numerical prediction of horizontal range agrees with theory, the trajectory of the puff front and the entrainment rate are found to differ significantly. Contrary to theoretical models and experiments, the DNS presented here accounts for the discontinuous injection that characterizes respiratory events where exhaled air accelerates until peaking and then decreases to the end of the cough. This laminar to turbulent transition over the jet-to-puff evolution, combined with the assumption of spherical puff topology and the turbulence role neglect under the linear momentum conservation premise, explain the shortcomings of the theoretical model in capturing the dynamics of thermal puffs produced by mildly violent respiratory events.

The authors report no conflict of interest.

## Data Availability

The data that support the findings of this study are available from the corresponding author upon reasonable request.
